# Outer membrane vesicles from flagellin-deficient *Salmonella enterica* serovar Typhimurium induce cross-reactive immunity and provide cross-protection against heterologous *Salmonella* challenge

**DOI:** 10.1038/srep34776

**Published:** 2016-10-04

**Authors:** Qiong Liu, Qing Liu, Jie Yi, Kang Liang, Bo Hu, Xiangmin Zhang, Roy Curtiss, Qingke Kong

**Affiliations:** 1 Institute of Preventive Veterinary Medicine, Sichuan Agricultural University, Chengdu, 611130, China; 2 Center for Infectious Diseases and Vaccinology, The Biodesign Institute, Arizona State University, Tempe, AZ, 85287-5401, USA; 3 Department of Medical Microbiology, School of Medicine, Nanchang University, Nanchang, 330006, China; 4 Department of Pathology and Laboratory Medicine, University of Texas Medical School at Houston, Houston, TX, 77030, USA; 5 Department of Pharmaceutical Sciences, Eugene Applebaum College of Pharmacy/Health Sciences, Wayne State University, Detroit, MI, 48202, USA

## Abstract

Outer membrane vesicles (OMVs) isolated from *Salmonella* Typhimurium are potentially useful for developing subunit vaccines because of high immunogenicity and protective efficacy. However, flagella might remain in OMV pellets following OMV purification, resulting in non-essential immune responses and counteraction of bacterial protective immune responses when developing a vaccine against infection of multiple serotypes *Salmonella*. In this study, a flagellin-deficient *S.* Typhimurium mutant was constructed. Lipopolysaccharide profiles, protein profiles and cryo-electron microscopy revealed that there were no significant differences between the wild-type and mutant OMVs, with the exception of a large amount of flagellin in the wild-type OMVs. Neither the wild-type OMVs nor the non-flagellin OMVs were toxic to macrophages. Mice immunized with the non-flagellin OMVs produced high concentrations of IgG. The non-flagellin OMVs elicited strong mucosal antibody responses in mice when administered via the intranasal route in addition to provoking higher cross-reactive immune responses against OMPs isolated from *S.* Choleraesuis and *S*. Enteritidis. Both intranasal and intraperitoneal immunization with the non-flagellin OMVs provided efficient protection against heterologous *S.* Choleraesuis and *S.* Enteritidis challenge. Our results indicate that the flagellin-deficient OMVs may represent a new vaccine platform that could be exploited to facilitate the production of a broadly protective vaccine.

Facultative intracellular pathogenic *Salmonella* spp. cause gastroenteritis and enteric fever, posing a hazard to human health and resulting in economic losses in animal industries^1^,^2^. With the emergence of multiple drug-resistant *Salmonella* strains, vaccination has begun to play an important role in controlling and preventing *Salmonella* infections around the world^1^,^3^. Killed vaccines and subunit vaccines of *Salmonella* provide short-term immunity and have been used with the variable efficacy, while attenuated live vaccines are associated with the potential risk of substantial infectivity in immunocompromised and elderly patients^4^. The ideal vaccine should fulfill a number of criteria, including protective efficacy and safety^5^.

Outer membrane vesicles (OMVs) are naturally produced by Gram-negative bacteria, such as *Escherichia coli*^6^, *Salmonella*^7^, *Helicobacter pylori*^8^ and *Neisseria meningitidis*^9^, and OMVs-like membrane vesicles are produced by Gram-positive bacteria, such as *Clostridium perfringens*^10^. OMVs range in average size from 20 nm to 500 nm. Additionally, OMVs from Gram-negative bacteria have a structure with a spherical bilayer membrane that contains biologically active components, such as membrane proteins and lipopolysaccharide (LPS). OMVs perform diverse biological functions, including the secretion and mediation of factors that contribute to bacterial pathogenicity^11^. LPS and outer membrane proteins (OMPs) contained in the OMVs may serve as adjuvants of immune- stimulating molecules to potently stimulate professional antigen-presenting cells (APCs) and thereby enhance immune responses^7^. OMVs are potential candidate targets for developing subunit vaccines and vaccine carriers because of their high immunogenicity and efficiency, lack of ability to infect hosts and their ability to deliver heterologous antigens^12^,^13^. A vaccine based on OMVs obtained from *N. meningitidis* that contains three highly immunogenic proteins has been approved for use in Europe in individuals over 2 months old^14^.

Following OMV purification, the OMV pellet can contain large macro-molecules, such as flagellin^15^. Flagellin, the structural protein contained in bacterial flagella, is common in pathogenic and commensal bacteria such as *Salmonella* and *E*. *coli*^16^,^17^. Flagellated *Salmonella*, which retain motility, can express two genetically distinct flagellin proteins, called phase 1 and phase 2 flagellin, which are encoded by the *fliC* and *fljB* genes, respectively^18^. The function of *Salmonella* flagella depends on the assembly of two monomeric flagellin proteins into a larger flagella polymer^16^. *Salmonella* flagellin elicits pro-inflammatory responses to epithelia through interactions with Toll-like receptor 5 (TLR5). The activation of TLR5 mobilizes nuclear factor (NF)-κB and induces the production of tumour necrosis factor-α (TNF-α)^19^. Therefore, flagellin is often used as an adjuvant in vaccines, in which the corresponding flagellin is engineered to display a foreign peptide on the surface, fused with foreign proteins, or chemically linked to bacterial polysaccharides^20^,^21^,^22^.

Flagellin acts as a good protective antigen because it is capable of providing protection against bacterial infection^23^,^24^. The presence of large amounts of flagellin in OMVs may interfere with the immunogenicity of other antigens or induce excessive pro-inflammatory responses as a result of the characteristics of the immune-stimulating molecule^16^,^25^. In this study, we analysed LPS profiles and identified proteins that were contained in OMVs purified from both a wild-type strain and a non-flagellin mutant. We then evaluated the structure of the purified OMVs using cryo-electron microscopy (EM). We also evaluated the immunogenicity and protective efficacy of the OMVs when administered via different routes of immunization. We demonstrated that the non-flagellin OMVs induced strong mucosal and systemic responses and provided effective protection against challenge with diverse *Salmonella* serovars.

## Materials and Methods

### Bacterial strains, plasmids and growth conditions

All strains and plasmids used in this study are listed in the Table 1. *S.* Typhimurium and *E. coli* were grown in Luria-Bertani (LB) broth or agar (Difco, Detroit, MI, USA) at 37 °C. LB agar containing 5% sucrose was used for *sacB* gene-based counter-selection during the construction of the mutant strain^26^. Diaminopimelic acid (DAP) (50 μg/ml) was added to the growth medium used for the ∆*asd* strains^27^. All experimental protocols were approved by Sichuan Agricultural University and Arizona State University.

The primers used in this study are listed in Table 2. DNA manipulations were performed as previously described^28^. *E*. *coli* were transformed using electroporation. Transformants were selected on LB agar plates containing the appropriate antibiotics. For construction of the ∆*fliC* mutation, two pairs of primers, fliC-1F/fliC-1R and fliC-2F/fliC-2R, were used to amplify approximately 300 bp upstream and downstream fragments of the gene *fliC*, respectively, from χ3761 (wild-type *S.* Typhimurium), and then the two fragments were joined by PCR using the primers fliC-1F/fliC-2R. The resulting PCR product had a terminal base A when using LA Taq enzyme (TaKaRa, Otsu, Japan), and it was then inserted to the T-terminal pYA4278 vector generated by AhdI digestion. This approach created the suicide plasmid pQK256 for *fliC* deletion. The same strategy was used to construct pQK257 for *fljB* gene deletion. ∆*fliC* and ∆*fljB* mutations were introduced into *S.* Typhimurium by a suicide plasmid method, generating mutant K83^29^.

### Purification and characteristics of OMVs

Unless otherwise specified, the OMVs described in this study are native OMVs (nOMVs) without any detergent processing, and isolated from *Salmonella* UK-1 and its mutants according to previously described methods^13^. Cryo-EM was used as previously described to evaluate the shapes of the purified OMVs^30^. The protein concentration in the OMVs was used to indicate OMV concentration in the following experiments. The protein concentration of the OMVs was measured using a bicinchoninic acid (BCA) assay (Thermo Pierce, Rockford, IL, USA), and 10 μg of each OMV sample, based on protein content, was analysed using SDS-PAGE and stained using GelCode^TM^ Blue Stain Reagent (Thermo Pierce, Rockford, IL, USA).

The LPS profiles of the OMVs were examined using standard methods^31^. The OMV samples were separated on 12% sodium dodecyl sulphate polyacrylamide gel electrophoresis (SDS-PAGE) gels and then visualized using silver staining. The quantification of LPS content in the same amount of each OMV sample (50 μg) was measured via Kdo (3-deoxy-D-manno-octulosonic acid) analysis. *S.* Typhimurium LPS (Sigma-Aldrich, Saint Louis, MO, USA) was used as the standard^32^.

For the LC-MS/MS analyses, each protein lane in the gel was cut into five slices, and each slice was then cut into 1 mm cubes. The procedures that were subsequently followed in these assays are described in a previous study^33^. Raw MS files were searched against *S.* Typhimurium LT2 in the Uniprot database (downloaded on February 14, 2016) using MaxQuant software. The precursor mass tolerance was set to 7 ppm. The fragment ion mass tolerance was set to 0.5 Da.

### Assays of OMV uptake and cytotoxicity assay in cell lines

OMVs were labeled and incubated with the 1% (vol/vol) lipophilic fluorophore dialkylcarbocyanine iodide (Dil) (Invitrogen, Grand Island, NY, USA) for 30 min at 37 °C. The OMV suspensions were ultracentrifuged (150,000 × g, 4 °C, 2 h) and washed three times with DPBS. Labeled OMV particles were pelleted in DPBS and determined the protein concentration to 2 mg/ml solutions by BCA protein assay kit (Thermo Pierce, Rockford, IL, USA) for use.

Murine macrophage RAW264.7 cells were maintained at 37 °C with 5% CO_2_ in DMEM (Gibco BRL, Grand Island, NY, USA) containing 10% FBS (Hyclone, Logan, UT, USA). Cells were plated onto the wells of a 24-well plate (5 × 10^5^ cells per well) covered with glass coverslips (Biokeystone, CA, USA) and cultured for 24 h. After three washed with PBS, labeled OMVs were added into culture medium at final concentration of 2 μg/ml. After culturing for 2 h, cells were washed three times with PBS and then fixed with 4% formaldehyde in PBS for 10 min, and later cells incubated with OMVs were blocked with SEA block buffer (Thermo Pierce, Rockford, IL, USA) for 1 h at room temperature. Nuclei were labeled with 4′, 6-diamidino-2-phenylindole (DAPI) (Invitrogen, Grand Island, NY, USA) for 30 min in room temperature. Cells were visualized with AMG EVOS digital inverted multi-functional microscope (Advanced Microscopy Group, Bothell, WA, USA) at 100 × magnification.

To investigate the cytotoxicity of OMVs for macrophages, diverse concentrations of OMVs ranging from 3.075 μg/ml to 100 μg/ml were added to RAW264.7 cells plated in 24-well plates (5 × 10^5^ cells/well). After 24 hours, the supernatants from each well were collected and evaluated by a Multitox-Fluor Multiplex Cytotoxicity Assay (Promega, Madison, WI, USA) according to the manufacturer’s instructions. All experiments were performed in triplicate.

### Ethic statement

All experiments involving animals were conducted in compliance with the Animal Welfare Act and related regulations of Sichuan Agricultural University related to animal experiments (Ya’an, China; Approval No. 2011-028). All animal works were approved by the committee of Sichuan Agricultural University. The principles stated in the Guide for the Care and Use of Laboratory Animals were followed. All efforts were made to minimize animal suffering during the experiments.

### Immunization and challenges in animal experiments

Female BALB/c mice (6 weeks old, 16–22 g) were purchased from the Dashuo Biotechnology Co., Ltd. (Chengdu, China). Mice were divided into groups of 5 or 6 and then intranasally immunized with 20 μg of OMVs suspended in 10 μl of Dulbecco’s Phosphate Buffered Saline (DPBS) buffer. Intraperitoneal immunizations were performed using 5 μg of OMVs suspended in 100 μl DPBS buffer. The intranasal administration of 10 μl of DPBS buffer or the intraperitoneal administration of 100 μl of DPBS buffer served as the negative controls for the respective immunization experiments. Booster immunizations were then given administered at 4 weeks after the first immunization. Blood samples were collected via orbital sinus puncture, and vaginal secretions were collected by repeatedly flushing the animals using 0.1 ml PBS buffer at intervals of two weeks. Following centrifugation, soluble fractions of sera and secretion samples were harvested and stored at −80 °C for future use. Five weeks after a booster immunization, the mice were challenged via an oral route with a lethal dose of the wild-type *Salmonella* strain in 20 μl of PBS with 0.01% gelatin (BSG buffer). The challenged mice were monitored daily for 30 days. The animal experiments were performed twice, and the data were combined for analysis.

### Enzyme-linked immunosorbent assay (ELISA)

*S.* Typhimurium LPS was purchased from Sigma-Aldrich Company (Saint Louis, MO, USA). OMPs were isolated from *Salmonella* as previously described^34^. Quantitative ELISA was used to analyse antibody levels. Briefly, solutions containing 2 μg of *Salmonella* OMPs or 1 μg of *Salmonella* LPS per well were suspended in 100 μl of sodium carbonate-bicarbonate coating buffer (pH 9.6) and used to coat NUNC MaxiSorp^TM^ 96-well plates (Thermo Scientific, Waltham, MA, USA). The plates were then incubated overnight at 4 °C. To obtain standard curves for each antibody isotype, plates were coated in triplicate with two-fold dilutions of the appropriate purified mouse Ig isotype standard IgG, IgG_1_, IgG_2a_ and IgA (BD Biosciences, San Jose, CA, USA), starting at 0.5 μg/μl. The plates were washed 3 times with PBST (PBS with 0.1% Tween 20) and then blocked with a 2% bovine serum albumin (BSA) solution for 2 h at room temperature. A 100 μl volume of suitably diluted sample was added in triplicate to the individual wells, and the plates were incubated for 1 h at room temperature. After washing the wells with PBST, biotinylated goat anti-mouse IgG, IgG_1_, IgG_2a_ and anti-IgA (Southern Biotechnology Inc., Birmingham, AL) was added to each well. The wells were then developed using a streptavidin-alkaline phosphatase conjugate (Southern Biotechnology, Inc., Birmingham, AL), and colour was detected using *p*-nitrophenylphosphate substrate (Sigma-Aldrich, St. Louis, MO, USA) in diethanolamine buffer (pH 9.8). Colour development (absorbance) was read at 405 nm using an automated ELISA plate reader (Bio-Rad iMark Microplate Reader, USA) after the appropriate incubation period. The final Ig isotype concentration in samples was calculated using appropriate standard curves, and a log-log regression curve was calculated from at least four dilutions of the isotype standards.

To confirm cross-reactivity between the OMVs from the flagellin-deficient mutant, serum IgG antibodies against OMPs from the heterologous serotypes *S.* Choleraesuis and *S.* Enteritidis were detected using competitive ELISA^35^. 2 μg per well of *S.* Typhimurium OMPs as the coated antigen were incubated in plates, and a 100 μl volume of 50-fold diluted serum sample was added to each well. After 1 h of incubation at room temperature, diluted *S.* Choleraesuis or *S.* Enteritidis OMPs as the competitive antigen (dilutions from 10-fold to 7,290-fold) were incubated in wells for 2 h at 37 °C. The next procedures were following our standard methods previously described. The absorbance at 405 nm was read in an automated ELISA plate (Bio-Rad iMark Microplate Reader, USA).

### Statistical analysis

One-way or two-way analysis of variance (ANOVA) was performed to determine the significance of differences between the mean values of the experimental and control groups. The data are expressed as the means ± standard deviations. The means were compared using the least significant difference test. The differences in survival rates among all groups were analysed using the log-rank sum test. *P* < 0.05 was considered a slight significant difference. All data were analysed statistically using the Graph-Pad Prism 5 software package (Graph Software, San Diego, CA, USA)^36^.

## Results

### Purification, characterization and visualization of OMVs

**Figure 1:**
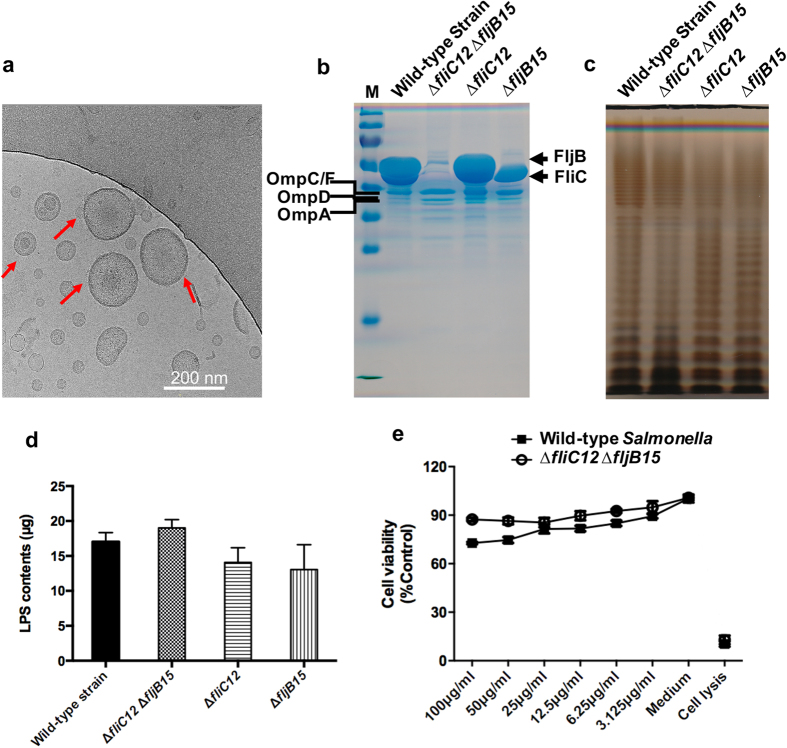
Characterization, visualization and cytotoxicity of OMVs derived from *S.* Typhimurium and non-flagellin mutant strain. (**a**) Cryo-EM imaging of OMVs. OMVs derived from the flagellin-deficient *S.* Typhimurium were visualized using cryo-EM. The red arrows indicate the visible OMVs. (**b**) In total, 10 μg of OMVs from each sample was subjected to 12% SDS-PAGE and stained with GelCode^TM^ Blue Stain. The major OMPs, including OmpA, OmpC/F and OmpD, are marked on the left, and the flagellar FliC and FljB proteins are labelled on the right. (**c**) LPS profiles from OMVs. LPS obtained from OMVs was visualized using silver staining after the samples were separated using 12% SDS-PAGE. (**d**) Quantification of LPS levels in OMVs. The same amount of OMVs (50 μg) was measured using a Kdo (3-deoxy-D-manno-octulosonic acid) analysis. *S.* Typhimurium LPS was used as the standard. (**e**) The cytotoxicity of OMVs derived from *S.* Typhimurium and the flagellin-deficient mutant in RAW264.7 macrophage cells. Cells were incubated with the corresponding OMVs at the indicated dose. Cell viability was determined by measuring the fluorescence in the supernatants using a Multitox-Fluor Multiplex Cytotoxicity Assay. Supernatants from cells without OMVs and cell lysis solution were treated to induce cell lysis, and these products were used as the negative and positive controls, respectively. Two-way ANOVA was performed to determine the significance of differences.

Three mutant strains, K081 (∆*fliC12*), K082 (∆*fljB15*) and K083 (∆*fliC12* ∆*fljB15*), and the wild-type strain *S.* Typhimurium χ3761 were used to purify OMVs (Table 1). Cryo-EM was used to evaluate the structure of the OMVs. OMVs purified from the mutant ∆*fliC12* ∆*fljB15* were spherical with a bilayer membrane, and no flagellin was visible (Fig. 1a). A large number of flagellin polymers were observed in the OMVs that were isolated from the wild-type strain, but the OMVs were still visible (Fig. S1).

Purified OMVs were analysed using SDS-PAGE and stained using GelCode^TM^ Blue Stain Reagent (Fig. 1a). Bands corresponding to both phase 1 and 2 flagellin were observed at approximately 50 kDa in the OMVs obtained from wild-type *S.* Typhimurium, while no bands corresponding to flagellin proteins were observed in the OMVs from the flagellin-deficient mutant. The major *Salmonella* OMPs OmpA, OmpD and OmpC/F were observed in all of the OMVs (Fig. 1). The MS analysis was also consistent with this result (Table 3). Among the proteins that were identified using MS, we also observed other effector proteins, such as SipABC and SopB in both of the OMV samples in addition to a large amount of OmpACD (Table 3). The LPS profiles and the quantification of the OMVs that were purified from the wild-type strain and its derivatives were also determined, and no obvious differences were observed following silver staining (Fig. 1c) or in a quantitative assay based on the Kdo method (Fig. 1d).

### Cytotoxic effects of OMVs in macrophages

To explore the effects of the OMVs on cell viability, we examined cytotoxicity in cells treated with the OMVs for 24 hours. Both the flagellin-deficient OMVs and the wild-type OMVs showed gradual cell lysis when inoculated with increased concentration ranging from 3.075 μg/ml to 100 μg/ml. The data showed that the viability of cells treated with the non-flagellin OMVs and with the wild-type OMVs did not have any significant difference regardless of the concentration of treated OMVs, indicating that the flagellin in the wild-type OMVs elicited only a mild level of cytotoxic activity (Fig. 1e)^16^,^37^.

### Uptake assay of OMVs in macrophages

**Figure 2:**
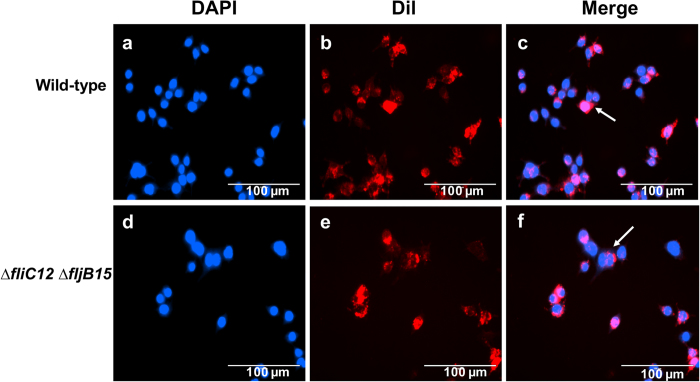
Internalization of OMVs by RAW264.7 macrophage cells. OMVs (2 μg/ml final concentration) were incubated with cells for 12 hours. OMVs were stained by 1% (vol/vol) lipophilic fluorophore dialkylcarbocyanine iodide (Dil) (red), and the nuclei (blue) were stained by DAPI. The results were recorded using an AMG EVOS digital inverted multi-functional microscope (AMG) at 100x magnification. Arrows indicate the internalization of OMVs derived from *S.* Typhimurium by macrophages.

As secretory vehicles for bacterial proteins and lipids, OMVs play critical roles in establishing colonization, transmitting virulence factors into host cells, and modulating host cell responses^11^,^38^. We determined the uptake of OMVs from the wild-type strain and the non-flagellin mutant by RAW264.7 macrophages. Microscopy analysis indicated that OMVs from the wild-type strain and the non-flagellin mutant strain were observed around the nucleus (blue), indicating that OMVs could be internalized by macrophages (Fig. 2), and the uptake of the different strains did not show obvious differences.

### OMVs elicit strong humoral and mucosal immune responses

To explore the immunogenicity of the OMVs from the flagellin-deficient mutant strain, groups of 6 mice each were intranasally or intraperitoneally immunized with the corresponding OMVs (20 μg in 10 μl DPBS or 5 μg in 100 μl DPBS, respectively). After immunization, all of the animals that were immunized via the intraperitoneal or intranasal route remained in good health and exhibited no abnormal behaviour.

**Figure 3:**
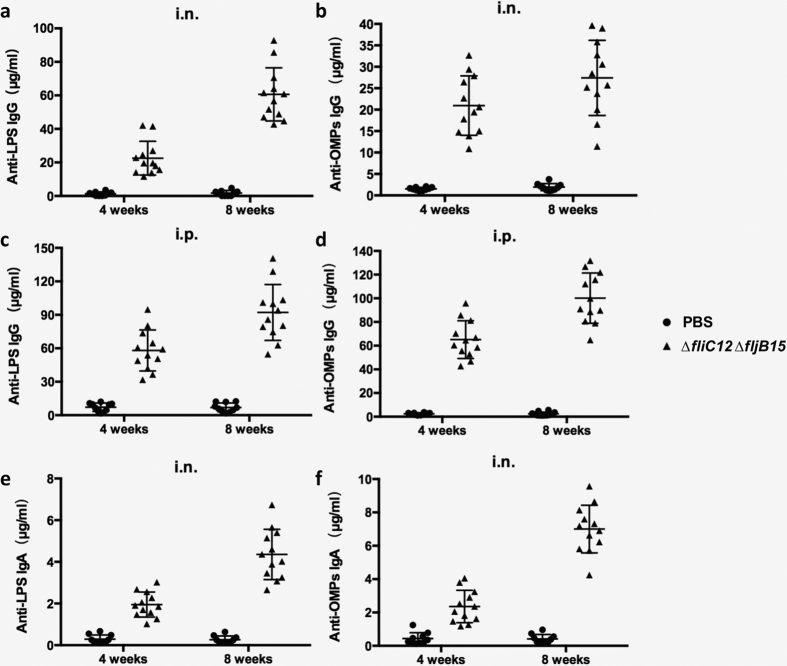
IgG and secretory IgA (S-IgA) immune responses were analysed in sera from mice immunized with OMVs. The total amount of anti-LPS (**a**) or -OMP (**b**) IgG in the sera obtained from mice immunized intranasally with OMVs, the total amount of anti-LPS (**c**) or -OMP (**d**) IgG in the sera obtained from mice immunized intraperitoneally with OMVs, and the total amount of S-IgA that was specific for LPS (**e**) or for OMPs (**f**) were measured using quantitative ELISA. Each group consisted of 10 (control) or 12 (vaccinated) mice. The mice were immunized with OMVs that were derived from *S.* Typhimurium and then boosted at week 5. Samples were collected at 4 weeks and 8 weeks after the first immunization. PBS-vaccinated mice served as the control group. The data shown represent the concentration of IgG or S-IgA antibodies in samples obtained from mice and are shown according to standard curves.

Quantitative ELISA was used to determine the levels of antibodies in the sera and vaginal washes that were obtained from the immunized mice. The results showed that the serum anti-LPS or anti-OMP IgG levels in the mice that were immunized intraperitoneally or intranasally with OMVs represented significantly higher levels than the control group (*P* < 0.05) at all time points (Fig. 3a–d). Furthermore, intraperitoneal immunization induced higher anti-LPS and anti-OMP IgG production than intranasal immunization despite the fact that the dose used for intraperitoneal immunizations was one-fourth of the dose used for intranasal immunizations (Fig. 3a–d).

We also detected the levels of secretory IgA (S-IgA) antibodies in vaginal washes that were collected from the mice immunized with OMVs. At 8 weeks after immunization, the anti-LPS or anti-OMP S-IgA levels in the samples obtained from mice that were intranasally immunized with OMVs were significantly higher than the control group (*P* < 0.05) (Fig. 3e,f). No S-IgA antibodies against either LPS or OMPs were detected in the vaginal secretions obtained from the mice intraperitoneally immunized at 8 weeks (data not shown).

**Figure 4:**
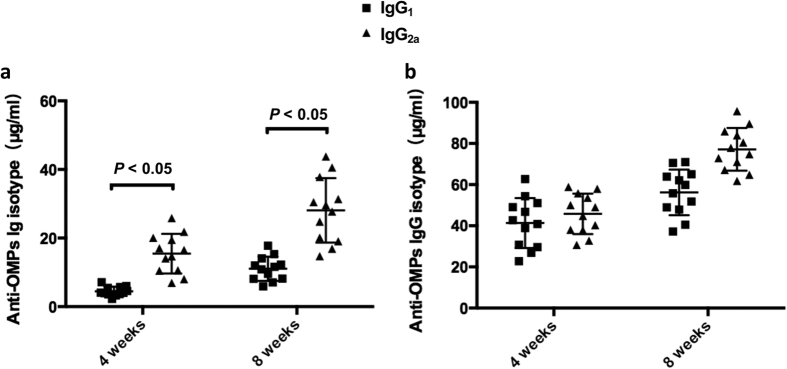
Serum IgG_1_ and IgG_2a_ responses in intranasally (**a**) and intraperitoneally (**b**) immunized and control mice. Outer membrane proteins (OMPs) as the coated immunogen. The data represented ELISA results determining the concentration of IgG_1_ and IgG_2a_ subclass antibody to OMPs in the serum of mice immunized by intranasal or intraperitoneal route with flagellin-deficient OMVs. Each group has 10 or 12 mice. Mice were boosted at week 5 and blood samples were collected on 4 weeks and 8 weeks after first immunization. PBS-vaccinated mice were in the control group. The error bars represented variations between triplicate wells.

The concentrations of antibody isotypes against OMPs, including IgG_1_ and IgG_2a_, were also measured by quantitative ELISA. The data showed that OMVs, administered with intranasal route, induced Th1-biased immune responses, as reflected by the significantly higher IgG_2a_ production (Fig. 4a). This effect was independent of the immunization route; however, compared to intranasal immunization, intraperitoneal immunization induced a trend towards balanced immune responses of IgG_1_ and IgG_2a_ (Fig. 4b).

### Protection against virulent *S.* Typhimurium challenge

**Figure 5:**
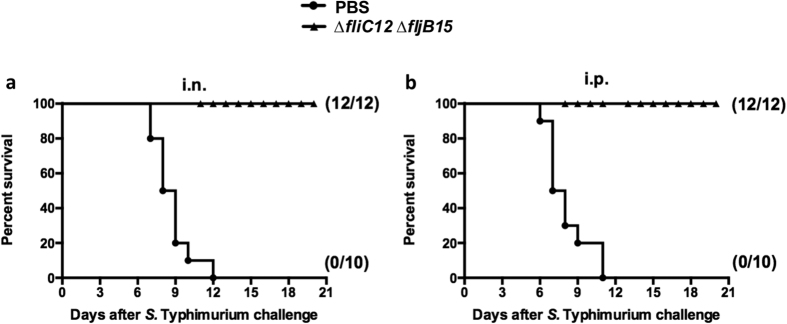
Survival in vaccinated mice after oral challenge with wild-type *S.* Typhimurium. Intranasal (**a**) and intraperitoneal (**b**) immunization with OMVs derived from *S.* Typhimurium flagellin-deficient mutants provided protection against oral challenge with wild-type *S*. Typhimurium in BALB/c mice. In total, 10 (control) or 12 (vaccinated) mice per group were immunized twice at 4-week intervals with the indicated OMVs. The mice were challenged with 10^9^ CFU of *S.* Typhimurium at 5 weeks after the boost immunization. Mortality was monitored for 3 weeks after challenge. The numbers in parentheses refer to the number of surviving mice and the total number of mice per group. All vaccine groups were significantly different from the PBS control group (*P* < 0.01).

Immunized mice were challenged via the oral route with 10^9^ CFU (2,000 × LD_50_) of the wild-type *S.* Typhimurium at 5 weeks after the booster immunization. Immunization with non-flagellin OMVs, administered either intranasally or intraperitoneally, provided 100% protection against oral *S.* Typhimurium challenge (Fig. 5). These results were significantly better than the survival rates in the PBS-immunized group (*P* < 0.05), all of which succumbed to challenge with the wild-type *Salmonella* within 12 days (Fig. 5).

### Evaluation of cross-reactive immune responses and protection against heterologous *Salmonella*

**Figure 6:**
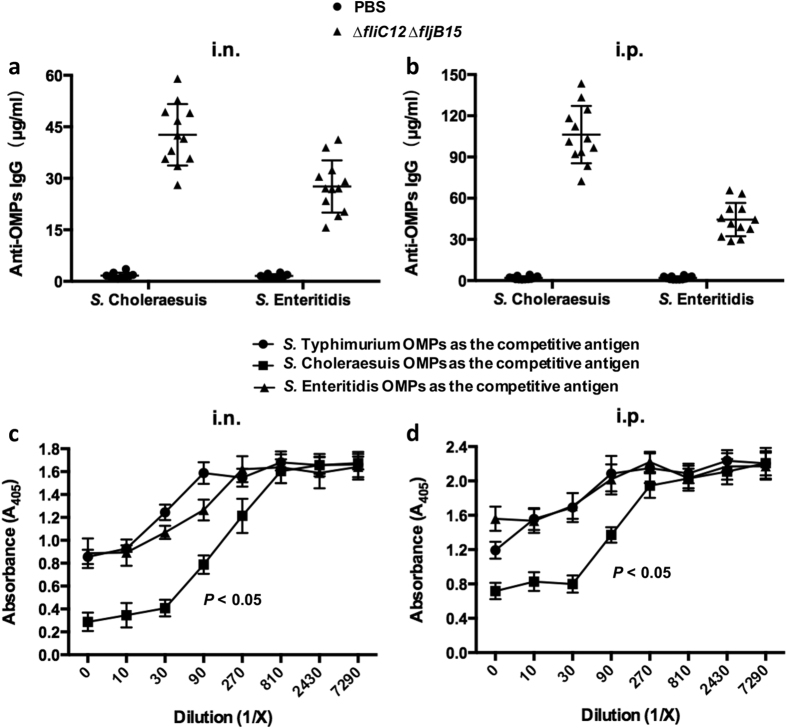
Cross-reactivity of OMVs derived from flagellin-deficient *S.* Typhimurium mutant strain. Cross-reactivity of IgG in sera obtained from intranasally (**a**) or intraperitoneally (**b**) immunized mice against OMPs from other serotypes of *Salmonella*, including *S.* Choleraesuis and *S.* Enteritidis, to analyse OMV-induced cross-protection. Each vaccinated group consisted of 12 mice, and the PBS group consisted of 10 mice. The cross-reactivity data represent the exact concentration of total IgG antibodies in the sera, as quantified using the corresponding standard curve using individual sera obtained from mice immunized intranasally or intraperitoneally with OMVs derived from *S.* Typhimurium. The error bars represent variations between triplicate wells. Competitive ELISA to determine the cross-reactivity of OMVs derived from flagellin-deficient mutant against heterologous *Salmonella.* OMPs isolated from *S.* Typhimurium were incubated into plates as coating antigen. OMPs from *S.* Choleraesuis, *S.* Enteritidis or *S.* Typhimurium (control) as the competitive antigen diluted from 1/10 to 1/ 7,290 were incubated in wells. The sera were obtained from mice (n = 10 or 12) immunized intranasally (**c**) or intraperitoneally (**d**) with OMVs at 8 weeks after the first immunization. The error bars represent variations from triplicate wells. *P* < 0.05 compared to the group of *S.* Typhimurium OMPs as the competitive antigen.

To investigate the ability of OMVs to induce cross-reactive immune responses against heterologous *Salmonella*, cross-reactive antibodies were evaluated using quantitative ELISA against OMPs that were isolated from *S.* Choleraesuis and *S.* Enteritidis. The flagellin-deficient OMVs that were administered either intranasally or intraperitoneally elicited significantly robust IgG levels against OMPs derived from the two *Salmonella* serovars compared with the control group (*P* < 0.05) (Fig. 6a,b). Moreover, the OMVs administered either intranasally or intraperitoneally induced higher IgG levels against the OMPs that were isolated from *S.* Choleraesuis than the levels against OMPs from *S.* Enteritidis (Fig. 6a,b). Furthermore, the competitive ELISA results further proved the cross-reactivity of non-flagellin OMVs against OMPs isolated from heterologous *Salmonella* serovars, and the cross-reactivity of non-flagellin OMVs against *S.* Choleraesuis OMPs was significantly higher these OMVs against OMPs from *S.* Enteritidis (*P* < 0.05) (Fig. 6c,d).

**Figure 7:**
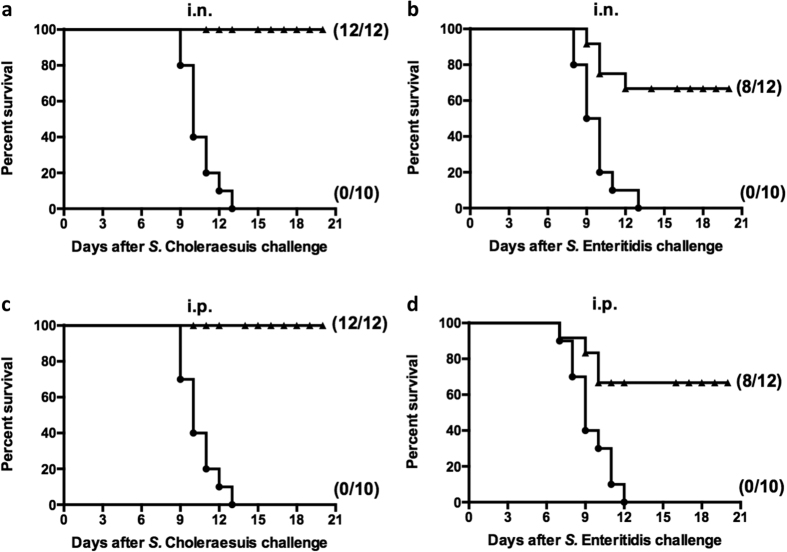
Cross-protection of OMVs derived from flagellin-deficient *S.* Typhimurium mutant strain. Immunized mice were challenged orally with 10^7^ CFU (~100-fold LD_50_) or 10^7^ CFU (~100-fold LD_50_) of wild-type *S.* Choleraesuis or *S.* Enteritidis, respectively. Mortality was monitored for 3 weeks after challenge. (**a**) Survival was followed in mice that were intranasally immunized using OMVs and subsequently submitted to by *S.* Choleraesuis challenge. (**b**) Survival was followed in mice that were intranasally immunized with OMVs and subsequently submitted to *S.* Enteritidis challenge. (**c**) Survival was followed in mice that were intraperitoneally immunized with OMVs and then submitted *S.* Choleraesuis challenge. (**d**) Survival was followed in mice that were intraperitoneally immunized with OMVs and then submitted to *S.* Enteritidis challenge. All vaccine groups were significantly different from the PBS control group (*P* < 0.01).

To examine the cross-protective ability of OMVs against other serotypes of *Salmonella* infection, immunized mice were orally challenged with 10^7^ CFU of *S.* Choleraesuis (~100 × LD_50_) or *S.* Enteritidis (~100 × LD_50_) at 5 weeks after the booster immunization. The flagellin-deficient OMVs that were administered either intranasally or intraperitoneally afforded 100% protection against *S.* Choleraesuis challenge (Fig. 7a,c). However, immunization with the non-flagellin OMVs provided 75% protection when mice were challenged with *S.* Enteritidis, and there was lower than the protective capacity conferred against wild-type *S.* Typhimurium (Figs 5 and 7b,d).

## Discussion

Compared to live attenuated vaccine candidates, OMVs provide several potential advantages, including a lack of replication, better safety, higher immunogenicity and intrinsic adjuvant effects that are provided by LPS, OMPs and other immune-stimulating molecules^11^,^38^. It is therefore of particular interest to develop subunit vaccines that are based on OMVs. For example, OMVs from *Neisseria lactamica*, *Pseudomonas aeruginosa*, *Vibrio cholerae* and *Clostridium perfringens* have been evaluated to determine their protective efficacy against the respective bacterial infections^9^,^10^,^15^,^39^, and one OMV-based vaccine has been licensed in Europe for the prevention of *N. meningitidis* infection in individuals over 2 months old^14^. OMVs obtained from *S*. Typhimurium induce protective immune responses and have been used as carriers to deliver antigens from other pathogens^13^,^40^. The major components of OMVs isolated from *Salmonella* are OMPs, which confer protection against lethal challenge with homologous *Salmonella* in mice^41^,^42^. It is therefore worthwhile to investigate the ability of OMVs to induce protective immunity against heterologous *Salmonella* serotypes.

Flagellin is highly immunogenic antigen capable of conferring protection against *Salmonella* infection. Both FliC and FljB flagellin proteins have a conserved region at the terminal ends essential for the assembly of the filament, and a variable central region accounting for antigenic variation with 114 H antigens presence in *Salmonella*^18^,^43^,^44^. While most of flagellin proteins are called FliC or FljB, but they don’t share the same antigenic determinants. For instance, FliC of *S*. Typhimurium possesses “i” H antigen, but FliC of *S*. Enteritidis has “g, m” H antigen^45^. These flagellins possessing distinct H antigen could not provide cross-protection against infection of heterologous *Salmonella*. And we observed a large amount of flagellin in the OMVs derived from the wild-type *Salmonella* (Fig. 1 and Fig. S1). These flagellin filaments in the OMVs may interfere with the immunogenicity of OMP and induce excessive pro-inflammatory responses^16^,^46^. Therefore, to develop a vaccine to confer broad protection against diverse *Salmonella* infection, the flagellin-deficient mutants were constructed by deletion of both the *fliC* and *fljB* genes. The ultrastructure of the OMVs that were obtained from *S.* Typhimurium was analysed using cryo-EM, and the results indicated the OMV sizes ranging from 20 to 250 nm (Fig. 1)^47^.

To develop an ideal, highly efficient vaccine for preventing *Salmonella* infection, both mucosal, systemic and cellular immunity should be considered^48^. Therefore, in this study we assessed mucosal and systemic immunogenicity of OMVs from flagellin-deficient *S.* Typhimurium when intranasally or intraperitoneally administered to mice. Consistent with previous studies^13^,^39^, mice intranasally immunized with OMVs showed high concentrations of secretory IgA in vaginal washes (Fig. 3), indicating that intranasal administration elicited robust mucosal immune responses, and flagellin-deficient OMVs may be a good candidate vaccine to induce strong mucosal immunity. We also observed that significantly higher levels of IgG were elicited against the OMPs in the mice immunized with OMVs by intraperitoneal route than the levels of IgG in the mice immunized by intranasal route despite that the dose of OMVs used for intraperitoneal immunizations was one-fourth of the dose of intranasal immunizations (Fig. 3a–d), the reason may probably rely on the facts that intraperitoneal immunization of *Salmonella* OMVs could more easily stimulate macrophage and dendritic cells and enhance production of pro-inflammatory mediators, thereby promoting the adaptive immunity^49^,^50^,^51^. We used a dose of 20 μg OMV for intranasal administration because high doses of antigen are required to either reach the intestinal tract or delivered directly by the posterior cervical lymph nodes^52^. Many studies have used even higher doses (>25 μg) of *Salmonella*-derived OMVs to immunize mice^7^,^13^. Consistent with previous studies showing that recombinant, bacterially derived OMVs have potential as a platform to induce a Th1-biased immune response^53^,^54^, immunity induced by intranasally administered OMVs resulted in a predominant Th1 response, while OMVs delivered by the intraperitoneal route induced a more balanced Th1/Th2 response (Fig. 4). Despite these subtle differences, both intranasal and intraperitoneal immunization routes conferred complete protection against wild-type *S.* Typhimurium challenge (Fig. 5). While it is generally believed that a Th1-dominated immune response is critical to achieve a protective immune response against *Salmonella* infection in mice^55^,^56^,^57^,^58^,^59^, mucosal immunity represented by production of secretary IgA on the mucosal surface is also considered to play an important role in preventing *Salmonella* colonization in the thick mucus layer of the intestinal epithelium^60^,^61^,^62^. Moreover, a previous study showed that intraperitoneal immunization with high doses (25 μg or more) of OMVs could represent a safety risk^63^. Therefore, intranasal immunization might be a more suitable strategy for vaccination with OMVs.

In this study, three *Salmonella* serotypes, which were isolated from commonly infected animals, were selected to evaluate the cross-protection conferred by OMVs. The results showed that OMVs derived from our Δ*fliC ΔfljB* mutant clearly induced the production of cross-reactive antibodies to OMPs from other *Salmonella* serovars (Fig. 6). The protective efficacy of the non-flagellin OMVs correlated well with the levels of antibodies that they induced (Figs 6 and 7). Mice immunized intranasally or intraperitoneally exhibited 100% protection following challenge with wild-type *S.* Choleraesuis, indicating that flagellin-deficient OMVs may be an advantageous, broadly protective vaccine that can prevent multiple types of *Salmonella* infections. At this point, it is not clear that the observed cross-protection can be attributed to the conserved OMPs or to other conserved antigens that are shared among these three *Salmonella* serovars, such as the LPS core oligosaccharide moiety that is possessed by all *Salmonella*^42^. Future research efforts should be directed towards up-regulating conserved OMPs by truncating the length of LPS from the core of the O-antigen unit, reducing endotoxic activity and increasing production of OMVs via the genetic engineering of *Salmonella* strains.

In summary, our study demonstrated that deletion of flagellin did not affect OMV production as reflected by an assay of LPS and proteins as well as by cryo-EM evaluation and LC-MS/MS analysis. Vaccination of mice using non-flagellin OMVs protected against challenge with *Salmonella* and the use of flagellin-negative OMVs further allowed for developing multi-serotype protective vaccines. These results provide a new platform to deliver other antigens from other pathogens and to develop broad-spectrum vaccines.

## Additional Information

**How to cite this article**: Liu, Q. *et al*. Outer membrane vesicles from flagellin-deficient *Salmonella enterica* serovar Typhimurium induce cross-reactive immunity and provide cross-protection against heterologous *Salmonella* challenge. *Sci. Rep.* **6**, 34776; doi: 10.1038/srep34776 (2016).

## Supplementary Material

Supplementary Information

## Figures and Tables

**Table 1:** Bacterial strains and plasmids used in this study.

Strain or Plasmid	Description	Source
Strains		
*S.* Typhimurium		
χ3761	*S.* Typhimurium UK-1	64
K081	∆*fliC12*	χ3761
K082	∆*fljB15*	χ3761
K083	∆*fliC12* ∆*fljB15*	K081
S100	*S.* Typhimurium, clinical isolate from duck	42
S246	*S.* Enteritidis, clinical isolate from chicken	42
S340	*S.* Choleraesuis, clinical isolate from pig	42
*E. coli*		
χ7232	*endA1 hsdR17 (rK−, mK+) supE44 thi-1 recA1 gyrArelA1Δ (lacZYA-argF) U169*λpir*deoR* (φ*80dlac Δ (lacZ) M15*)	65
χ7213	thi-1 thr-1 leuB6 glnV44 tonA21 lacY1 recA1 RP4-2-Tc :: μλpir Δ*asdA4* Δ*zhf-2* :: Tn 10	65
Plasmids		
pYA4278	Suicide vector, *sacB mobRP4 oriR6K* Cm^r^	26
pQK256	For deletion of *fliC*	This study
pQK257	For deletion of *fljB*	This study

**Table 2:** Primers used in this study.

Primer	Sequence (5′-3′)	Function
fliC-1F	CGTTCTTTGTCAGGTCTGTC	For deletion of
fliC-1R	GATTAGCGGCCGCGATCTTTTCCTTATCAATTA	*fliC* by suicide
filC-2F	AAGATCGCGGCCGCTAATCCGGCGATTGATTCAC	plasmid
filC-2R	TGTACCCGGCACAGACGGTC	
fljB-1F	AGTGAGCTCCACGTTCATGT	For deletion of
fljB-1R	AATTAGCGGCCGCAAAATTTTCCTTTTGGAAGG	*fljB* by suicide
fljB-2F	ATTTTGCGGCCGCTAATTTATTTCGTTTTATTC	plasmid
fljB-2R	GTCATTACCTGATAATTCTTC	

**Table 3:** Major proteins identified from flagellin-deficient and wild-type OMVs.

UniProt accession	MW (kDa)*	Protein name	Subcellular localization	Intensity**	Matched peptides
OMVs isolated from flagellin-deficient *Salmonella*					
O30916	61.934	Inositol phosphate phosphatase (SopB)	Periplasmic	1.42E + 10	63
P0CL52	73.941	Cell invasion protein (SipA)	Extracellular	1.23E + 10	68
P0CL47	42.983	Cell invasion protein (SipC)	Extracellular	1.16E + 10	39
P02936	37.515	Outer membrane protein A (OmpA)	Outer Membrane	9.83E + 09	24
P0A263	41.238	Outer membrane protein C (OmpC)	Outer Membrane	7.12E + 09	26
P37592	39.679	Outer membrane porin protein (OmpD)	Outer Membrane	7.05E + 09	22
Q56019	62.45	Cell invasion protein (SipB)	Extracellular	4.49E + 09	39
Q7CQN4	8.3914	Major outer membrane lipoprotein 1 (Lpp1)	Outer Membrane	4.00E + 09	7
P16328	49.834	Flagellar hook-associated protein 2 (FliD)	Extracellular	3.93E + 09	43
P02941	59.613	Methyl-accepting chemotaxis protein II (Tar)	Cytoplasmic Membrane	2.59E + 09	27
Q7CP97	65.491	Fumarate reductase flavoprotein subunit (FrdA)	Cytoplasmic Membrane	1.55E + 09	34
P26466	50.548	Maltoporin (LamB)	Outer Membrane	1.55E + 09	20
P0A1J5	59.109	Flagellar hook-associated protein 1 (FlgK)	Extracellular	1.53E + 09	35
Q8ZRP0	89.525	Outer membrane protein assembly factor (BamA)	Outer Membrane	1.52E + 09	49
Q8ZLZ4	53.685	Outer membrane channel (TolC)	Outer Membrane	1.48E + 09	27
Q7CQW9	18.865	Tol protein required for outer membrane integrity (Pal)	Outer Membrane	1.44E + 09	8
P0A1×0	15.547	Outer membrane lipoprotein (SlyB)	Outer Membrane	1.33E + 09	10
P35672	61.795	Protein (InvG)	Outer Membrane	1.10E + 09	37
Q8ZQT5	46.148	Protein (TolB)	Periplasmic	1.09E + 09	20
Q7CQP6	27.992	Scaffolding protein for murein-synthesizing holoenzyme (MipA)	Outer Membrane	1.02E + 09	11
OMVs isolated from the wild-type *Salmonella*					
P06179	51.611	Flagellin (FliC)	Extracellular	1.33E + 10	48
P0CL47	42.983	Cell invasion protein SipC	Extracellular	8.83E + 09	35
P0CL52	73.941	Cell invasion protein SipA	Extracellular	5.01E + 09	61
Q56019	62.45	Cell invasion protein SipB	Extracellular	3.66E + 09	45
P52616	52.535	Phase 2 flagellin (FljB)	Extracellular	3.33E + 09	40
P16328	49.834	Flagellar hook-associated protein 2 (FliD)	Extracellular	2.12E + 09	38
P0A263	41.238	Outer membrane protein C (OmpC)	Outer Membrane	1.67E + 09	21
Q7CQD4	26.448	Guanine nucleotide exchange factor (SopE2)	Extracellular	1.19E + 09	16
P74873	60.047	Secreted effector protein (SptP)	Extracellular	9.38E + 08	31
Q8ZQC8	37.782	Secreted effector protein (SopD2)	Cytoplasmic	7.07E + 08	17
Q56026	37.112	Cell invasion protein (SipD)	Extracellular	6.88E + 08	24
P16326	34.175	Flagellar hook-associated protein (FlgL)	Extracellular	6.56E + 08	21
P02936	37.515	Outer membrane protein A (OmpA)	Outer Membrane	6.40E + 08	19
Q8ZNR3	86.782	E3 ubiquitin-protein ligase (SopA)	Periplasmic	6.00E + 08	36
P37592	39.679	Outer membrane porin protein (OmpD)	Outer Membrane	5.76E+08	16
Q7CQW9	18.865	Tol protein required for outer membrane integrity (Pal)	Outer Membrane	1.41E + 08	8
Q7CQVB	18.494	Outer membrane protease, receptor for phage (OmpX)	Outer Membrane	1.41E + 08	11
Q93GL9	26.169	Conjugative transfer: surface exclusion (TraT)	Outer Membrane	8.42E + 08	7
Q8ZP50	22.958	Outer membrane protein (OmpW)	Outer Membrane	7.44E + 08	5
Q7CQP6	27.992	Scaffolding protein for murein-synthesizing holoenzyme (MipA)	Outer Membrane	7.44E + 08	11

^*^The values represented as MW (kDa) indicate the calculated molecular weight of the identified proteins.

^**^This table lists the 20 proteins with the highest abundance that were identified in the flagellin-deficient and wild-type OMVs in the end arrangement.
