# Supplementary material for: Outer membrane vesicles from flagellin-deficient Salmonella enterica serovar Typhimurium induce cross-reactive immunity and provide cross-protection against heterologous Salmonella challenge
Source: Sci Rep. 2016 Oct 4;6:34776. doi: 10.1038/srep34776 (PMC5048178; doi:10.1038/srep34776)
Supplement: Supplementary Information [file srep34776-s1.pdf]

Outer membrane vesicles from flagellin-deficient *Salmonella enterica* serovar Typhimurium induce cross-reactive immunity and provide cross-protection against heterologous *Salmonella* challenge

Qiong Liu[a, b, c, #], Qing Liu[a, #], Jie Yi[a], Kang Liang[a], Bo Hu[d], Xiangmin Zhang[e], Roy Curtiss III[b], Qingke Kong[a, b, *]

Affiliation

[a] Institute of Preventive Veterinary Medicine, Sichuan Agricultural University, Chengdu, China, 611130

[b] Center for Infectious Diseases and Vaccinology, The Biodesign Institute, Arizona State University, Tempe, AZ, USA, 85287-5401

[c] Department of Medical Microbiology, School of Medicine, Nanchang University, Nanchang, China, 330006

[d] Department of Pathology and Laboratory Medicine, University of Texas Medical School at Houston, Houston, TX, USA, 77030

[e] Department of Pharmaceutical Sciences, Eugene Applebaum College of Pharmacy/Health Sciences, Wayne State University, Detroit, MI, USA, 48202

[#]Both authors contributed equally to this research

[*] Corresponding author: Qingke Kong

Email address: kongqiki@163.com

Figure S1. Cryo-EM imaging of OMVs. OMVs derived from wild-type *S.* Typhimurium (A) and the flagellin-deficient mutant (B) were visualized by cryo-EM. The red arrows indicate the visible OMVs.

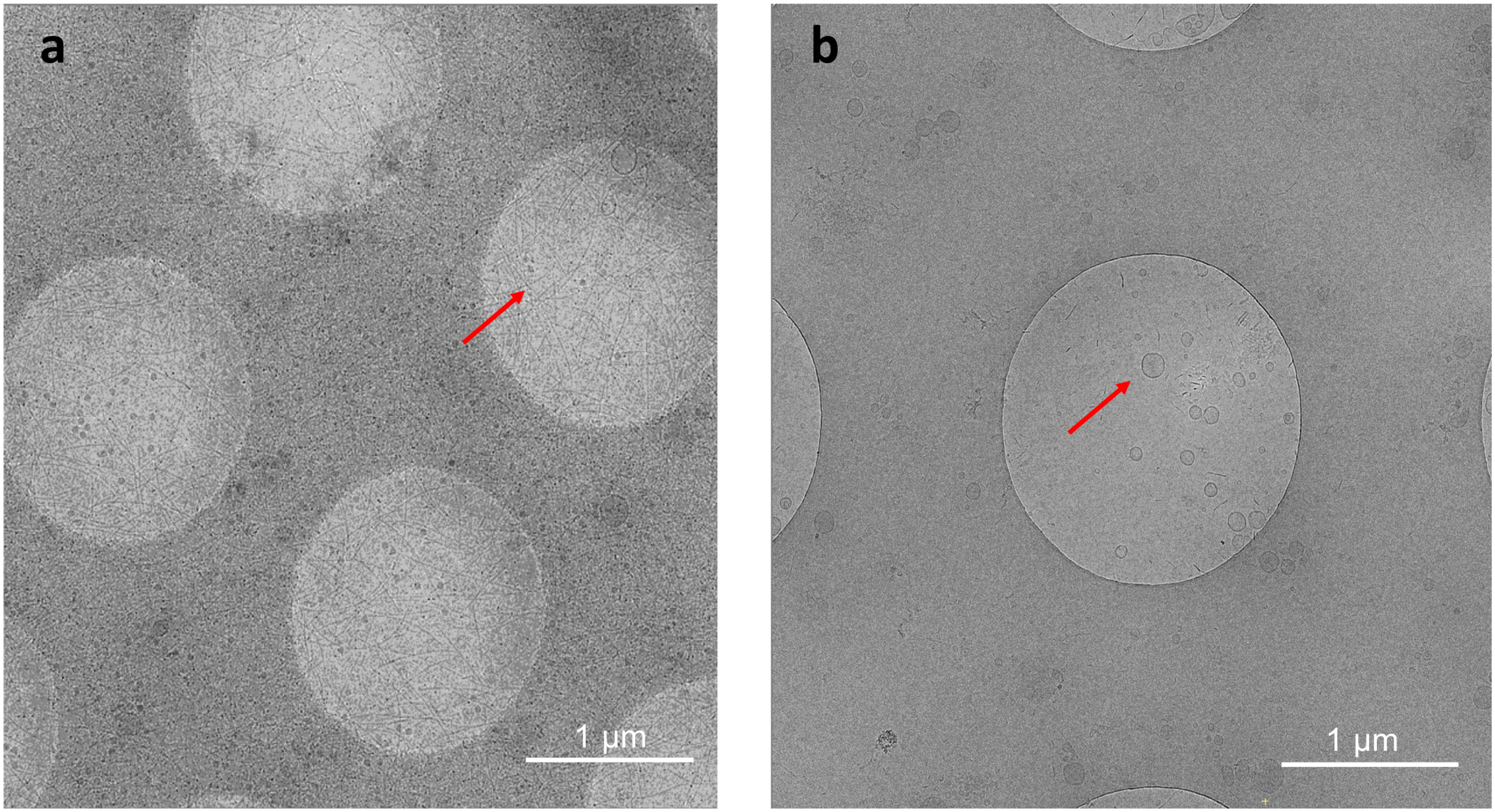
a
1 µm
b
1 µm
